# Unveiling the drivers of geographic atrophy progression in eyes with reticular pseudodrusen

**DOI:** 10.1371/journal.pone.0329907

**Published:** 2025-08-12

**Authors:** Mingui Kong, So Young Han, Sungsoon Hwang, Ga-In Lee, Je Moon Yoon, Don-Il Ham

**Affiliations:** 1 Department of Ophthalmology, Kangbuk Samsung Hospital, Sungkyunkwan University School of Medicine, Seoul, Korea; 2 Department of Ophthalmology, Samsung Medical Center, Sungkyunkwan University School of Medicine, Seoul, Korea; 3 Department of Ophthalmology, Hangil Eye Hospital, Incheon, Korea; 4 Department of Ophthalmology, Catholic Kwandong University College of Medicine, Incheon, Korea; National Eye Institute, UNITED STATES OF AMERICA

## Abstract

**Purpose:**

To evaluate the progression rate of geographic atrophy (GA) and identify factors associated with GA expansion in eyes with reticular pseudodrusen (RPD) using fundus autofluorescence (FAF) photography.

**Methods:**

A total of 28 eyes from 28 patients diagnosed with GA and RPD, who completed a 3-year follow-up, was included. The eyes underwent thorough examination with color fundus photography, FAF and near infrared (NIR) imaging, and spectral domain optical coherence tomography (SD OCT). The areas of atrophic lesions were quantified using FAF images at years 1, 2, and 3 employing a semi-automated software (Region Finder).

**Results:**

The rate of increase in mean GA area in eyes with RPD was calculated to be 0.39 ± 0.21 mm per year over the 3-year period. The annual progression rate measured over three years showed a statistically significant increase each year. Univariate analysis revealed that subfoveal choroidal thickness (SFCT) and RPD distribution were independently associated with GA progression (P ≤ 0.037). In multivariate analysis, thin SFCT was the only significant factor linked to an increased rate of GA growth. The progression from extrafoveal GA to foveal GA was solely associated with the initial square root GA area.

**Conclusion:**

These results offer valuable insights for the development of interventional strategies aimed at mitigating GA enlargement in this specific subgroup of patients.

## Introduction

Geographic atrophy (GA) constitutes 35% to 40% of late age-related macular degeneration (AMD) cases [1]. GA is linked with delayed dark adaptation, decreased contrast sensitivity, and diminished reading ability. It eventually leads to central vision loss when the fovea is affected [2–4]. While effective treatments for GA were previously lacking, recent reports have highlighted FDA-approved therapies that slow down its progression [5,6]. This has spurred increased interest in comprehending GA progression. Given the reported efficacy of FDA-approved treatments in inhibiting GA progression, a thorough analysis of the actual rate of GA progression becomes a pivotal area of investigation.

Color fundus photography (CFP) has traditionally been a mainstay imaging modality for GA diagnosis; however, it has limitations in precisely delineating GA boundaries. In addition to CFP, other imaging techniques such as fundus autofluorescence (FAF) and optical coherence tomography (OCT) are now employed for measuring GA area [7]. FAF imaging, a noninvasive technique, relies on retinal fluorophores, primarily lipofuscin of the ocular fundus [8]. In areas of GA, concurrent lipofuscin depletion due to loss of retinal pigment epithelium (RPE) results in a markedly reduced autofluorescent signal [9]. Leveraging this contrast difference between GA and non-atrophic areas, Region Finder (Spectralis HRA + OCT; Heidelberg Engineering, Heidelberg, Germany), a semi-automated software, can identify and quantify GA areas with high reliability and reproducibility [10,11].

Reticular pseudodrusen (RPD), also called subretinal drusenoid deposits, are yellowish-white networks of interlacing round to oval drusenoid deposits. They were first described in 1990 by Mimoun, [12] as yellowish interlacing patterns in the fundus, most apparent in red-free and blue light. Recently, RPD have been identified as risk factors for the progression of late AMD, [13,14] particularly GA [15–18]. Despite previous studies detailing the natural history, progression, and predictive factors of GA, [19–22] limited information exists regarding GA progression, especially in eyes with RPD. Although RPD has been repeatedly identified as an important factor associated with rapid progression of GA, [23,24] its specific characteristics and mechanisms remain largely unknown. Therefore, in this study, we focused exclusively on eyes with GA in the presence of RPD, using FAF images and the Region Finder software for analysis. Through this approach, we aimed to identify specific features within RPD that are strongly associated with rapid GA progression, contributing to a deeper understanding of its role as a high-risk factor.

## Method

This study was conducted in accordance with the principles of the Declaration of Helsinki and received approval from the Institutional Review Board of Samsung Medical Center, Seoul, Korea (IRB file number 2016-01-017). We conducted a retrospective analysis of medical records of patients previously diagnosed with RPD at Samsung Medical Center between January 2010 and December 2012. Given the retrospective design of this study and the use of anonymized data, requirements for informed consent were waived by the institutional review board IRB at the Samsung Medical Center in Korea. The data were accessed for research purposes from January 20, 2016, to May 20, 2016. The authors did not have access to information that could identify individual participants. Eyes diagnosed with RPD were included if they were also diagnosed with GA and underwent examinations with a minimum of 3-year follow-up. If both eyes of a patient met the inclusion criteria, the eye with the larger GA area was selected for the study.

Exclusion criteria comprised any ophthalmologic conditions that could potentially impact the diagnosis of RPD and GA (such as laser scars, retinal vein occlusion, central serous chorioretinopathy, hypertensive retinopathy, and diabetic retinopathy), any indication or history of wet AMD including choroidal neovascularization, polypoidal choroidal vasculopathy, and retinal angiomatous proliferation, a prior history of intraocular procedures, including cataract surgery, performed within the 6 months leading up to enrollment or during the study duration, incomplete examinations (e.g., absence of any OCT or multimodal fundus images, or incomplete follow-up), or eyes with lesion borders extending beyond the 30-degree field used for FAF imaging.

### Ocular examination and image acquisition

A thorough ophthalmologic assessment was performed, encompassing slit-lamp biomicroscopy, indirect ophthalmoscopy, CFP with blue-channel imaging, near-infrared (NIR) imaging, red-free photography, fundus autofluorescence imaging, fluorescein angiography (FA), indocyanine green angiography (ICGA), and spectral-domain optical coherence tomography (SD OCT). CFP images were acquired using a Topcon IX50 camera (Topcon, Paramus, NJ, USA) and subsequently reviewed using the Topcon ImageNet software (version 2.56; Topcon) to evaluate the blue-channel details, following previously established protocols [25]. Additionally, red-free images, near-infrared photographs, autofluorescence imaging, SD OCT scans, FA, and ICGA studies were conducted with the Spectralis HRA + SD OCT system (Heidelberg Engineering, Heidelberg, Germany) and interpreted using Heidelberg’s proprietary analysis software. OCT images were acquired using the Spectralis HRA + OCT system (version 1.7.0.0; Heidelberg Engineering, Heidelberg, Germany). A raster scan was conducted on all eyes in the study, centered at the fovea, following the enhanced depth imaging (EDI) protocols. The raster scan comprised 31 B-scans, each containing 768 A-scans, with a length of 9.0 mm and spaced 240 μm apart, covering a 30 degree x 25 degree area. The automatic real-time (ART) mode utilizing the eye-tracker system was employed, averaging a total of 25 frames for each B-scan image. During the OCT examination, subjects were instructed to maintain fixation on the internal fixation target. Additionally, horizontal and vertical EDI OCT cross-sectional scans at the fovea were performed for all subjects. Subfoveal choroidal thickness (SFCT) was manually measured at the foveal center using the software provided with the SD OCT device, extending from the outer portion of the hyperreflective line corresponding to the retinal pigment epithelium to the inner surface of the sclera.

### Diagnosis of reticular pseudodrusen

The diagnosis of RPD followed the criteria established in previous studies [16]. Specifically, RPD was diagnosed based on multimodal imaging findings. Diagnostic criteria included: (1) multiple yellowish white lesions with a reticular pattern on CFP; (2) a light, interlacing network on red-free imaging; (3) hyporeflective lesions with mild background hyperreflectance on NIR; (4) hypofluorescent lesions against a mildly hyperfluorescent background on FAF; (5) subretinal deposits on SD OCT; and (6) hypofluorescent lesions in the mid-to-late phase of ICGA. On SD OCT, RPD lesions were defined as five or more hyperreflective mounds or triangular lesions above the RPE, observed on more than one B-scan. RPD was considered definite when lesions were identified on at least three imaging modalities, including SD OCT. The distribution of RPD was assessed by the extent of retinal involvement according to previously described criteria. FA and ICGA were only performed for differential diagnosis with other lesions such as cuticular drusen.

To evaluate the distribution of RPD, we utilized color fundus montages constructed from five individual images: one centered on the macula and four surrounding fields (superior, inferior, temporal, and nasal). These images were captured using a 50-degree fundus camera (IX50, Topcon). The resulting montage enabled visualization of the RPD distribution extending into the mid-peripheral retina. Based on the extent and area of involvement, RPD distribution was classified into three categories: localized, intermediate, and diffuse, following previously established criteria (S1 Fig) [26,27].

### Definition of GA, imaging processing, grading and analysis

**Fig 1:**
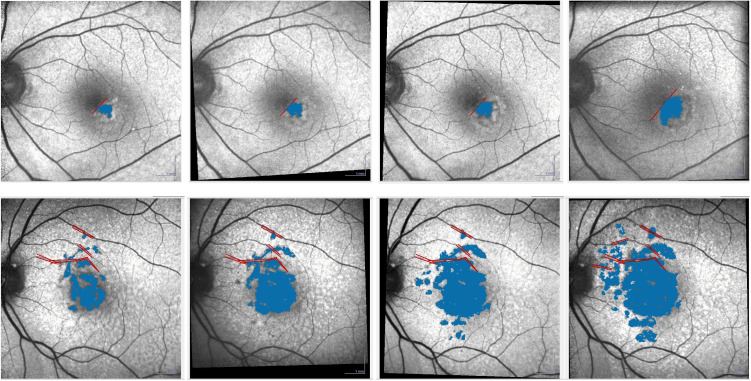
Measurement of GA area using the Region Finder software (Heidelberg Engineering, Heidelberg, Germany). The GA area was increased every year. The red lines are manual constraint that the reader could use to perform measurement more precisely. The automatic algorithm could not go over the delimited zone.

FAF imaging was performed with an excitation wavelength of 488nm and an emission range of 500–700nm, centered on the macula with a field of view set at 30 degrees by 30 degrees with high resolution (1536 x 1536 pixels). Each FAF image was created from a minimum averaging of 10 frames. GA was defined as a sharply demarcated lesion with clearly reduced FAF, measuring at least 0.05mm^2^ or more (approximately 178μm in diameter), and not associated with wet AMD. Measurement of GA area was conducted using the Region Finder software (version 2.5.7.0) as described previously, [10,11] by two independent readers (M.K. and S.H.) with disparities <15% using the average of the two measurements from both readers (Figure 1). If the difference was ≥ 15%, a third expert (D.-I.H) measured the GA area. The latter measurement, along with the closer one of the two readers’ measurements, was then averaged to obtain the final value. Patterns of increased FAF were independently determined by two other observers (G.-I.L and J.M.Y) using a previously reported classification method [28]. In cases of disagreement, a senior observer (D.-I.H) served as an arbitrator.

Lesion progression rate was defined as the change in lesion size between two observation times: from baseline to year 1, from year 1 to year 2, and from year 2 to year 3. The mean growth rate over the 3-year period was calculated. In the analysis of GA progression rate, both GA area and the square root of GA area were utilized for analysis to minimize the effect of baseline lesion size.

### Statistical analysis

Data were analyzed using Wilcoxon signed rank test, Friedman test and Kendall test for comparison of GA progression rate among years. Univariate and multiple regression with backward stepwise method was conducted for univariate and multivariate analysis, respectively. Pearson’s correlation coefficient (rho) was used when both variables were continuous. Logistic regression analysis was utilized to identify factors associated with the progression of extrafoveal GA to foveal GA. A P-value < 0.05 was considered as statistically significant. All statistical analyses were performed using SPSS 23.0 software.

## Results

Out of 43 eyes from 43 patients diagnosed with both RPD and GA, with completion of a 3-year follow-up, 28 eyes were included in this study. Fifteen eyes were excluded for the following reasons: Two eyes developed wet AMD during follow-up, 4 eyes had diabetic retinopathy, 1 eye had retinal vein occlusion, and 1 eye required surgical intervention during the study period due to vitreous hemorrhage caused by a retinal tear. Additionally, 7 eyes exhibited GA borders extending beyond the 30-degree field used for FAF imaging. The mean age of the 28 included patients was 73.7 ± 7.1 years (range, 60–92 years). Their mean best-corrected visual acuity (BCVA) was 0.20 ± 0.18 on the logarithm of the minimum angle of resolution (LogMAR) scale. The mean SFCT was 138.1 ± 45.6 μm. Twenty-seven (96.4%) of the 28 patients were females. Among the 28 eyes, 17 (60.7%) had accompanying typical drusen. Based on the standard types of RPD distribution previously described, [26] 1 eye (3.6%) was classified as localized type, 8 eyes (28.6%) as intermediate type, and 19 eyes (67.8%) as diffuse type (Table 1). The mean GA area and square root of GA area at baseline were 2.49 ± 2.84 mm^2^ and 1.29 ± 0.91 mm, respectively. All analyses related to GA area were conducted using only the square root of the GA area. Five eyes (17.9%) were categorized in the unilobular group, while 23 eyes (82.1%) were in the multilobular group. Foveal GA was present in 5 eyes (17.9%), while 23 eyes (82.1%) exhibited extrafoveal GA. Over the 3-year follow-up, extrafoveal GA progressed to involve the fovea in 8 eyes (34.9%) out of the 23 eyes initially presenting with extrafoveal GA. The distribution of eyes by increased FAF pattern is presented in Table 1.

**Table 1:** Clinical characteristics of subjects, geographic atrophy and reticular pseudodrusen at baseline.

		RPD without late AMD	
Number of eyes		28	
Age		73.7 ± 7.1 (range, 60–92)	
Gender		27/1 (female/male)	
VA (LogMAR)		0.20 ± 0.18	
SFCT (*μ*m)		138.1 ± 45.6	
Accompanying typical drusen		17 (60.7%)	
GA area (mm^2^)		2.49 ± 2.84 (range, 0.05-8.73)	
Square root of GA (mm)		1.29 ± 0.91 (range, 0.22-2.95)	
GA morphology		Unilobular	5
		Multilobular	23
GA type		Foveal	5
		Extrafoveal	23
FAF pattern		None	4
		Focal	10
		Patch	2
		Band	1
		Diffuse	11
RPD Distribution	Localized	1 (3.6%)	
	Intermediate	8 (28.6%)	
	Diffuse	19 (67.8%)	

RPD, reticular pseudodrusen; AMD, age-related macular degeneration; VA, visual acuity; LogMAR, logarithm of the minimum angle of resolution; SFCT, subfoveal choroidal thickness; GA, geographic atrophy; FAF, fundus autofluorescence.

**Fig 2:**
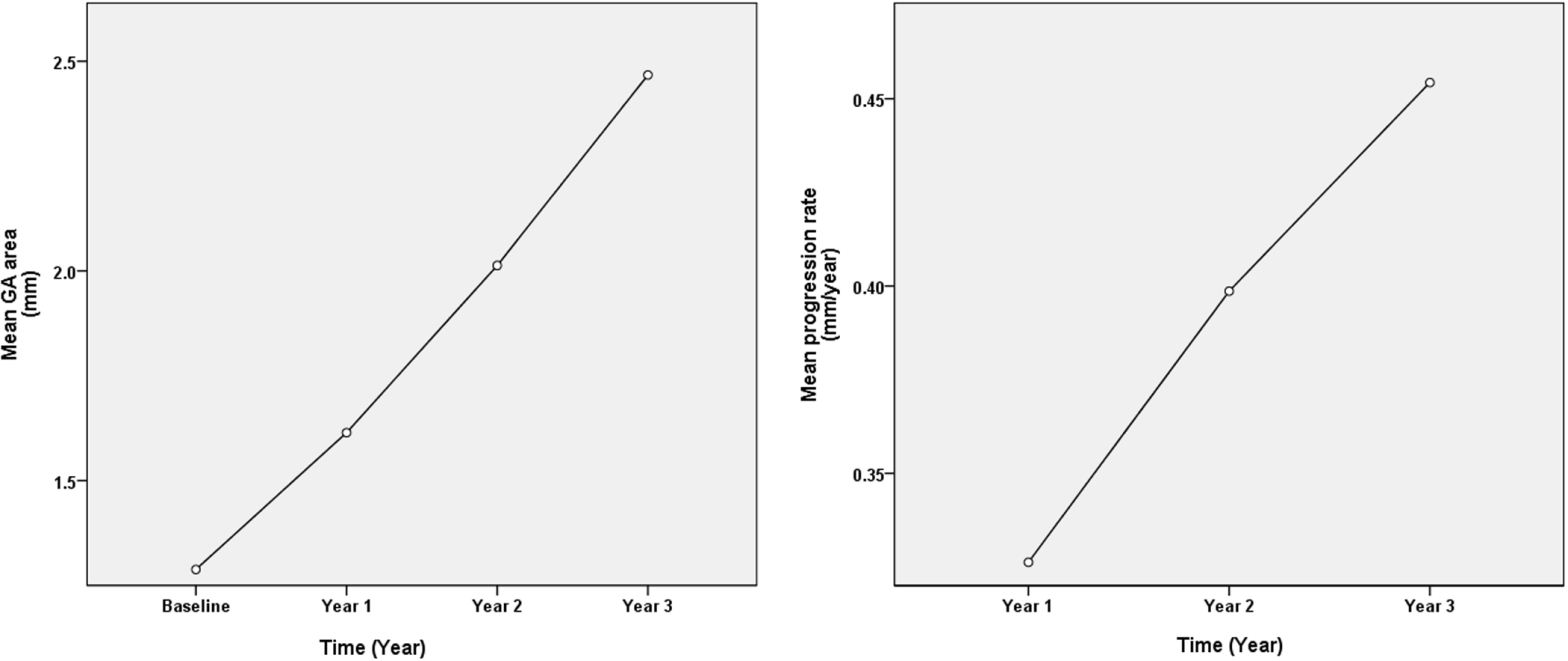
Mena GA area (mm) and mean GA progression rate (mm/year) over time (year).

The mean progression area of square root of GA in eyes with RPD over the 3-year period was 0.39 ± 0.21 mm/year. The rate of atrophy enlargement was slowest in year 1 (0.33 ± 0.24 mm/year), followed by year 2 (0.40 ± 0.34 mm/year), and year 3 (0.45 ± 0.23 mm/year). This progression rate demonstrated a statistically significant acceleration over the 3-year period. (Table 2, Fig 2).

**Table 2:** Progression rate of geographic atrophy area by time.

	Year 1	Year 2	Year 3	Overall	P value^*^
Rate of progression (mm/year)	0.33 ± 0.24	0.40 ± 0.34	0.45 ± 0.23	0.39 ± 0.21	**0.019**

P-value among year 1, 2, and 3.

In the univariate analysis, baseline factors significantly correlated with the mean GA growth rate over the 3 years included SFCT (rho = −0.416, P = 0.028), and RPD distribution (P = 0.037, Table 3). In the multivariate analysis, thin SFCT was identified as the sole factor associated with the rate of GA area progression (Table 4). Initial square root GA area was the exclusive factor linked to the progression from extrafoveal GA to foveal GA (P = 0.042).

**Table 3:** Progression rate of geographic atrophy area by various factors (univariate analysis).

Factors at baseline	Mean progression rate of GA (mm/year)	
	rho	P value
Age	0.142	0.471
BCVA	0.097	0.622
SFCT	−0.416	0.028*
Accompanying typical drusen	N/A	0.096
GA area (mm)	0.248	0.202
GA morphology	N/A	0.141
GA type	N/A	0.758
FAF pattern	N/A	0.790
RPD distribution	N/A	0.037*

rho, Pearson correlation coefficient; BCVA, best corrected visual acuity; SFCT, subfoveal choroidal thickness; GA, geographic atrophy; FAF, fundus autofluorescence; RPD, reticular pseudodrusen; * statistically significant.

**Table 4:** Progression rate of geographic atrophy area by various factors (Multivariate analysis).

Factors at baseline	Mean progression rate of GA area (mm/year)		
	B ± SE	Partial R^2^	P value
SFCT	−0.02 ± 0.01	0.173	0.028

B, coefficients; SE, standard error; R2, coefficient of determination; SFCT, subfoveal choroidal thickness; GA, geographic atrophy.

## Discussion

Various factors related to GA progression have been reported in a range of studies. Researches utilizing OCT have indicated that early complete retinal pigment epithelial and outer retinal atrophy (cRORA) progresses more slowly compared to advanced lesions [29,30]. The ARMS2/HTRA1 genotype has been associated with GA progression and initial GA enlargement, though not with multifocality [23,31]. An intriguing development is recent research suggesting that RPD is a risk factor for GA progression [23]. In this study, the presence of RPD was found to be associated with rapid GA progression independently of the ARMS2/HTRA1 genotype, though its relationship with foveal involvement remains unclear.However, limited information exists on factors influencing changes in GA progression rates over time, especially in eyes with RPD. In this study, a statistically significant trend of acceleration was observed over the three-year period. While comparisons between years 1 and 2 (p = 0.202) and years 2 and 3 (p = 0.084) were not significant, the difference between years 1 and 3 was statistically significant (p = 0.026). These findings support the importance of long-term follow-up for assessing GA progression. A previous study with shorter follow-up durations (six months or more) may have yielded different results due to the gradual nature of GA progression [32].

The mean lesion progression rate in this study was 0.39 ± 0.21 mm/year over three years, which is comparable to the rate reported in previous studies (0.379 mm/year) that highlighted rapid progression in eyes with RPD and in those with ARMS2/HTRA1 genotypes [23,31]. Given the observed acceleration over time, longer-term studies may report even higher rates. Differences in GA progression rates across studies could also stem from the inclusion of diffuse trickling-type GA, a subtype often linked to RPD and known for its rapid progression. Moreover, the exclusion of eyes with GA extending beyond the 30-degree imaging field (unmeasurable with Region Finder) may have influenced variability in reported rates. Large-scale, long-term studies using broader classifications and wide-field imaging may better elucidate these differences.

Various prognostic markers can affect GA progression such as baseline GA size, [33] previous progression rate, [34] bilaterality of GA lesion, [35] increased FAF pattern [36] and some genotypes [21].

This study aimed to explore whether particular RPD features contribute to GA progression or whether RPD itself is associated with faster GA growth. Lesion size, SFCT, FAF pattern, and GA morphology (unilobular or multilobular) were linked to GA progression in RPD eyes. However, FAF pattern may reflect baseline atrophy extent and thus be confounded by lesion size [19]. In our analysis, GA area, SFCT, FAF pattern, and morphology were not significantly correlated (P ≥ 0.345), so were not considered confounders. Thin SFCT has been associated with GA in multiple studies [37,38]. It is also commonly observed in eyes with RPD [39,40]. In this study, thin SFCT and diffuse RPD distribution were identified as factors associated with GA progression in univariate analysis, and these two variables were significantly related (P = 0.035). Although thin SFCT and diffuse RPD distribution were both associated with GA progression, these variables were significantly related, and the nature of their interrelationship makes it difficult to determine whether their contributions are independent. Further investigation is warranted to clarify whether RPD-associated GA progression is primarily mediated by underlying choroidal thinning or involves separate mechanisms.

Additionally, it is important to consider conflicting findings from other studies. For instance, a recent OCTA study by Wu et al. reported that the presence of RPD in eyes with intermediate AMD was not associated with significant impairments in choriocapillaris flow or choroidal vascular structure. These discrepancies may reflect differences in the stage of the maculopathy, as noted in the previous report, and highlight the importance of considering disease stage when interpreting the relationship between RPD and choroidal changes [41]. A Japanese study reported significantly faster GA progression in eyes with RPD (0.34 ± 0.17 mm/year) versus those without RPD (0.15 ± 0.13 mm/year, p < 0.001), and in eyes with a thin choroid compared to pachychoroid eyes (0.27 ± 0.18 mm/year vs. 0.11 ± 0.07 mm/year, p < 0.001) [24]. Future studies including eyes with thin choroid but without RPD may help clarify their independent contributions to GA progression.

In this study, GA progression from extrafoveal lesion to foveal lesion occurred in 34.9% of eyes with extrafoveal GA during 3 years. This was higher than that (31% in 4 years) in eyes with AMD, including typical drusen, pigmentary disturbance and/or RPD, reported in a previous study using CFP [42]. There could be a chance of underestimation in detecting GA by using CFP. In detection of GA lesion and measurement of the area, FAF images are superior to CFP, especially for eyes with multifocal GA lesions since lesion clarity and contrast on FAF images are better than those on CFP [22]. CFP has limitations in identifying GA areas and precisely outlining boundaries of lesions, and thus has less accurate quantification over time [35]. There are differences in mean lesion size measurement using FAF imaging compared to CFP [22]. Considering multilobular form of GA is far more commonly seen in RPD, [43,44] using FAF images on measuring GA area in eyes with RPD is necessary.

In our analysis, initial GA area was the only factor associated with foveal involvement. Previous studies have identified additional risk factors such as outer retinal thickness, distance to the fovea, and the thin double-layer sign [45]. Notably, one study even suggested that the double-layer sign might serve as a protective factor against foveal involvement [46]. These differing findings underscore the complexity of GA progression and the need for further research to clarify these relationships.

The proportion of female participants in our study was 96.4%, higher than previous reports of female predominance in RPD (61–87%) [47]. Notably, a previous study on RPD conducted by the same research team reported a female predominance of 86.2%, [47] consistent with earlier findings. Given these observations, further large-scale studies could be helpful to explore a potential association between female sex and GA progression in RPD.

This study has several limitations. First, this was a retrospective study, and a notable number of eyes were excluded due to various reasons. Second, we did not evaluate the effect of the following variables: body mass index (BMI), smoking status, or genotype. However, it has been reported that BMI or smoking history has no impact on GA growth [42]. In previous studies, genotype has been reported as a factor influencing GA progression independently of RPD [21,23,31]. Third, we did not differentiate between cRORA and advanced GA since we analyzed using FAF images. Thus, we did not evaluate ellipsoid zone disruption as a predictive variable in this study. However, because outer retina disruption exists in various degrees in eyes with RPD, we conducted our analysis using FAF instead of OCT [48–50]. Fourth, this study is limited by the use of a qualitative approach to assess the distribution of RPD. While this method aligns with current clinical practice, it may lack the precision and reproducibility offered by quantitative image analysis or AI-assisted evaluation. Future studies employing such advanced techniques may yield more detailed and objective insights into the clinical relevance of RPD distribution. Lastly, the exclusion of eyes in which GA progression extended beyond the 30-degree field used for FAF imaging could be a potential limitation. In cases of GA associated with RPD, the atrophic area may widely extend beyond the vascular arcades, surpassing the 30-degree field. Utilizing wider-field FAF imaging, such as 50-degree or greater, could provide a more comprehensive and accurate assessment of GA progression in these eyes, offering deeper insights into its natural course. Despite these limitations, we diagnosed RPD using SD OCT as well as multimodal images, which allowed accurate detection of RPD [51]. Using the Region finder software, a reliable tool for identification and quantification of even mulilobular GA lesions was also a strength of this study.

In conclusion, the current study demonstrated that the area of GA increased over time, with an accelerated rate of progression observed during follow-up. Both thin SFCT and diffuse distribution of RPD were associated with lesion progression, although their interrelationship limits conclusions about their independent effects. While treatment response was not evaluated, these findings may aid in stratifying risk and improving our understanding of disease progression in eyes with RPD-associated GA.

## Supporting information

S1 Fig Fundus montages of 3 distribution types. (A) Localized distribution. Reticular pseudodrusen (RPD) are observed in the central field and less than 1/3 area of superior and temporal photographic fields. (B) Intermediate distribution. RPD are observed in the central field, more than 1/3 area of superior field, and less than 1/3 area of temporal field. (C) Diffuse distribution. RPD are observed in the central field and more than 1/3 area of all 4 adjacent fields taken by the protocol. (Reprinted with permission from Lee MY, Yoon J, Ham D-I: Clinical features of reticular pseudodrusen according to the fundus distribution. Br J Ophthalmol 2012 Sep;96(9):1222-6. Copyright BMJ Publishing Group LTD.).
(TIF)
